# Retinal Microvascular Abnormalities and Risk of Renal Failure in Asian Populations

**DOI:** 10.1371/journal.pone.0118076

**Published:** 2015-02-06

**Authors:** WanFen Yip, Charumathi Sabanayagam, Boon Wee Teo, Wan Ting Tay, M. Kamran Ikram, E. Shyong Tai, Khuan Yew Chow, Tien Y. Wong, Carol Y. Cheung

**Affiliations:** 1 Singapore Eye Research Institute, Singapore National Eye Center, Singapore, Singapore; 2 Department of Ophthalmology, Yong Loo Lin School of Medicine, National University of Singapore and National University Health System, Singapore, Singapore; 3 Office of Clinical Sciences, Duke-NUS Graduate Medical School, Singapore, Singapore; 4 Department of Medicine, Yong Loo Lin School of Medicine, National University of Singapore and National University Health System, Singapore, Singapore; 5 Memory Aging & Cognition Centre, National University Health System, Singapore, Singapore; 6 National Registry of Diseases Office, Singapore, Singapore; Queen’s University Belfast, UNITED KINGDOM

## Abstract

**Background:**

Retinal microvascular signs may provide insights into the structure and function of small vessels that are associated with renal disease. We examined the relationship of retinal microvascular signs with both prevalent and incident end-stage renal disease (ESRD) in a multi-ethnic Asian population.

**Methods:**

A total of 5763 subjects (aged ≥40 years) from two prospective population-based studies (the Singapore Malay Eye Study and the Singapore Prospective Study) were included for the current analysis. Retinopathy was graded using the modified Airlie House classification system. Retinal vascular parameters were measured using computer-assisted programs to quantify the retinal vessel widths (arteriolar and venular caliber) and retinal vascular network (fractal dimension). Data on ESRD was obtained by record linkage with the ESRD cases registered by National Registry of Diseases Office, Singapore. Multi-variable adjusted regression analyses were performed to assess the associations of baseline retinal vascular parameters and prevalent and incident ESRD.

**Results:**

At baseline, 21(0.36%) persons had prevalent ESRD. During a median follow-up of 4.3 years, 33 (0.57%) subjects developed ESRD. In our analyses, retinopathy was associated with prevalent ESRD (multi-variable adjusted odds ratio [OR], 3.21, 95% confidence interval [CI]: 1.28–8.05) and incident ESRD (multi-variable adjusted hazard ratio [HR], 2.51, 95%CI: 1.14–5.54). This association was largely seen in person with diabetes (HR, 2.60, 95%CI: 1.01–6.66) and not present in persons without diabetes (HR, 1.65, 95%CI: 0.14–18.98). Retinal arteriolar caliber, retinal venular caliber and retinal vascular fractal dimension were not associated with ESRD.

**Conclusion:**

Retinopathy signs in persons with diabetes are related to an increased risk of ESRD; however, other microvascular changes in the retina are not associated with ESRD.

## Introduction

Renal disease, particularly end-stage renal disease (ESRD), is a costly and disabling condition with a high mortality rate. [1] The pathological processes underlying the development of ESRD are not well understood. [2,3] Microvascular alterations including hyalinosis and muscular hyperplasia [4] in the renal microvasculature are common histopathological findings in individuals with ESRD. [5] These microvascular abnormalities have been suggested to represent early pathological abnormalities in the kidney. [5] However, such microvascular changes occurring in the glomerular vascular bed cannot be visualised directly and non-invasively. [6]

Since the retinal and renal circulations share similar anatomic and physiologic characteristics, [7–9] the retinal microvasculature provides an opportunity to study the renal microvasculature non-invasively. Microvascular changes in the retina such as the diameter of retinal vessels can now be quantitatively measured from retinal photographs. Several previous cross-sectional studies have documented that these microvascular changes (retinal arteriolar narrowing, presence of retinopathy signs, abnormal retinal vascular network) are associated with renal impairment. [6,10–12] There are fewer prospective studies investigating the relationship between retinal microvascular abnormalities and renal impairment with less consistent findings. [13–17] For example, in the Beaver Dam Chronic Kidney Disease study, authors did not find any statistically significant association between retinal vessel diameters (retinal arteriolar narrowing and venular widening) and the decline in eGFR over time (S1 Table). This discrepancy may be attributed to the use of different surrogate markers for renal impairment, age distributions and ethnicity across populations. [13–16] Importantly, none of the previous studies have examined the association with ESRD, the advanced form of renal disease. There have also been no prior studies examining these relationships in Asian populations, even though Asians have different risk factors for renal impairment compared to the Western populations. [18,19]

In this study, we examined the relationship of retinal microvascular signs with both prevalent and incident ESRD in a multi-ethnic Asian population.

## MATERIALS AND METHODS

### Study population

The present study utilized data from the Singapore Prospective Study Program (SP2) and The Singapore Malay Eye (SiMES) study. Both studies were combined to increase the number of incident ESRD cases in examining the relationship between retinal parameters and incident ESRD cases. Participants from both SP2 and SiMES cohorts were examined in the same study clinic (Singapore Eye Research Institute), following standardized clinical and retinal photographic protocols, except that blood samples were collected in non-fasting state in SiMES and fasting state in SP2. Details of both study participants and methods have been described elsewhere. [12,20]

In brief, the Singapore Prospective Study Program (SP2), included participants from one of four previous cross-sectional studies: Thyroid and Heart Study 1982–1984, [21] National Health Survey 1992, [22] National University of Singapore Heart Study 1993–1995 [23] or National Health Survey 1998. [24] All studies involved a random sample of individuals from the Singapore population, aged 24–95 years. From 2003 to 2007, 5157 participants attended the clinical examination and 4137 were offered retinal photography. Retinal photographs were available for 4098 participants. We excluded participants, who were younger than 40 years of age and those who were non Chinese, Malay or Indian, those with ungradable retinal photographs leaving 3163 for the final analysis. Written informed consent was obtained from each participant; the study was conducted according to the Declaration of Helsinki. Ethical approval was obtained from the Institutional Review Boards of the National University of Singapore and Singapore General Hospital.

The Singapore Malay Eye Study (SiMES), a population-based cross-sectional study of eye diseases in urban Malay adults ranging in age between 40 and 80 years residing in south-western Singapore, for this analysis. In brief, participants were selected, using an age-stratified (by 10-year age group) random sampling method; of 4168 eligible participants, 3280 participated in the study, conducted from August 2004 through June 2006. The methodology and objectives of the study population have been reported in detail elsewhere. [25] We excluded participants with ungradable retinal photographs leaving 3274 for the final analysis.

Written informed consent was obtained from each participant; and the study was conducted according to the Declaration of Helsinki. Ethical approval was obtained from the Institutional Review Boards of the Singapore Eye Research Institute.

### Retinopathy Signs

**Fig 1:**
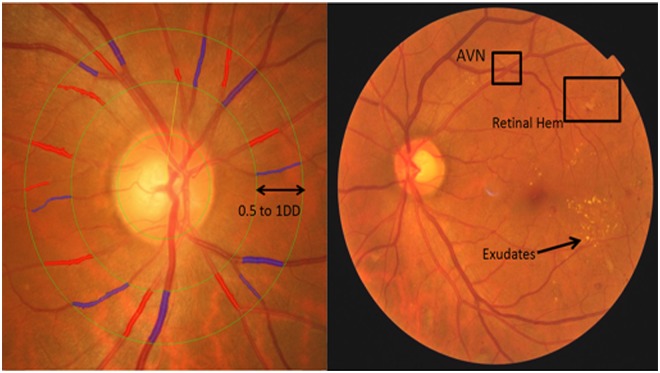
Retinal vascular calibre measurement and Retinopathy. Retinal arteriolar and venular calibers were summarized as central retinal arteriolar (CRAE) and the central retinal venular (CRVE) equivalent respectively from retinal fundus photograph using the Interactive Vessel Analysis software (IVAN, University of Wisconsin, US). Arterioles are in red and venules are in blue. Retinopathy was considered present if any characteristic lesion (microaneurysms, haemorrhages, cotton wool spots, intraretinal microvascular abnormalities, hard exudates, venous beading and new vessels) was present. [30]

Retinopathy was considered present if any characteristic lesion (microaneurysms, haemorrhages, cotton wool spots, intraretinal microvascular abnormalities, hard exudates, venous beading and new vessels) was present (Fig. 1). [21][19] For each eye, a retinopathy severity score was assigned accordingly and retinopathy was defined as being present if the retinopathy score (a scale modified from the Airlie House classification system) was at level 15 or higher [26].

### Retinal vascular calibre measurement

Retinal fundus photographs of both eyes were taken after dilating the pupils with 1% tropicamide and 2.5% phenylephrine hydrochloride, using a digital non-mydriatic retinal camera (CR-DGi with a 10D SLR backing; Canon, Tokyo, Japan). Two retinal images of each eye were obtained, one centered at Early Treatment for Diabetic Retinopathy Study (ETDRS) standard field 1 (the optic disc) and another centered on the ETDRS standard field 2 (the fovea). [27,28] Trained graders, masked to the participants’ characteristics, used a computer-based program, Interactive Vessel Analysis software (IVAN) program (University of Wisconsin, US), [29] to measure [20]retinal vascular caliber. Retinal vascular caliber was measured through a specified zone of 0.5 to 1 disc diameter away from the optic disc margin (Fig. 1). Optic disc-centered image of the right eye for most participants were analyzed, and the left eye in those without gradable right eye images. Based on the revised Knudtson-Parr-Hubbard formula [30] retinal arteriolar and venular calibers were summarized as central retinal arteriolar equivalent (CRAE) and central retinal venular equivalent (CRVE), respectively. Arterio-venous ratio (AVR) was not used in our study, as it cannot specify whether a change in AVR value is due to generalized arteriolar narrowing, venular dilation, or both. [31–33] Reproducibility of retinal vascular measurements was high, with intra-grader intraclass correlation coefficients [95% confidence interval (CI)] 0.99 (0.98–0.99) for CRAE and 0.94 (0.92–0.96) for CRVE. [12]

### Retinal vascular fractal dimension measurement

**Fig 2:**
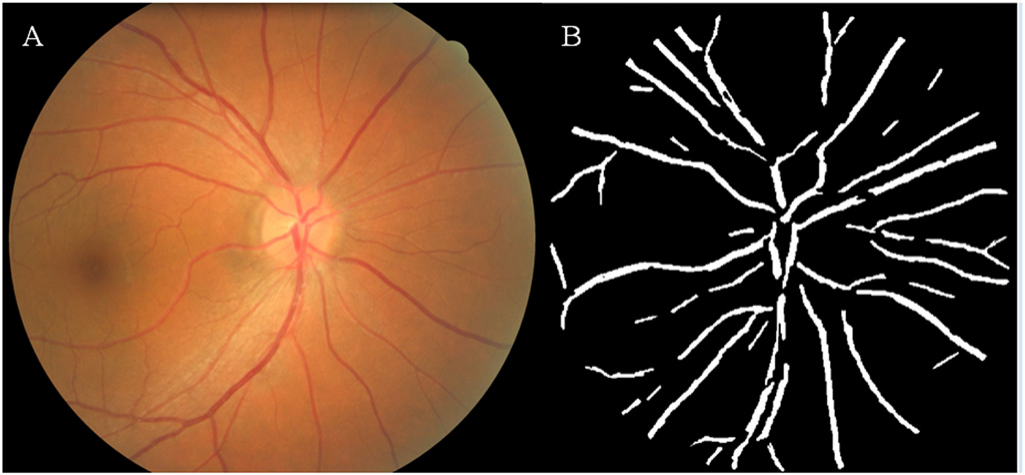
Retinal vascular fractal dimension measurement. A) Coloured fundus image B) Assessment of retinal vascular fractal dimension using the International Retinal Imaging Software—Fractal (IRIS-FRACTAL).

Fractal analysis was performed from the optic disc centered retinal photographs. Retinal images from the right eye were analyzed, unless they were ungradable, in which case, the left eye retinal images were used. Trained graders, masked to participants’ characteristics, used a computer-based program [International Retinal Imaging Software (IRIS-Fractal)] for fractal analysis of the photographs based on a standardized protocol described in an earlier trial. [34] The fractal dimension of the retinal vasculature was measured within a predefined circular area centered on the optic disc of 3.5 disc radii (Fig. 2). After all the retinal vessels within this region were automatically traced by IRIS-Fractal, the grader compared the tracing with the photograph and deleted artifacts which were mistakenly identified as vessels, such as peripapillary atrophy, retinal pigment abnormalities, choroidal vessels and reflection from the nerve fiber layer. Subsequently, fractal analysis was performed by the program, and the fractal dimension was calculated using the box counting approach. [35] The intra-grader intraclass correlation coefficients of IRIS-Fractal measurements were ranged from 0.93 to 0.95 [34].

### Prevalent ESRD Outcome

Prevalent ESRD was defined by the Registry as (a) the Glomerular Filtration Rate (corrected to the body surface area of 1.73m^2^) of the patient is less than 15 ml/min; or (b) the serum creatinine level of the patient is more than or equal to 5.7mg/dl; or (c) the patient’s kidney function has deteriorated to the extent that, the patient requires treatment for kidney failure. [36]

### Incident ESRD Outcome

Incident ESRD from all causes was obtained by linking with the ESRD cases registered by National Registry of Diseases Office, Singapore, by record linkage. The Register electronically captures new cases via a unique identifier number given to all Singapore citizens and residents (National Registration Identity Card). The Registry is expected to capture all ESRD cases in Singapore. New ESRD cases are identified by the Registry through various sources including notifications by healthcare professionals, hospital data for cases admitted to hospitals, and pathology and laboratory reports. The Registry manages the database with quality assurance to ensure that all data collected for the registries have been validated and are properly anonymized before analysis. [37,38]

New ESRD cases were identified by the Registry and defined as (a) the Glomerular Filtration Rate (corrected to the body surface area of 1.73m^2^) of the patient is less than 15 ml/min; or (b) the serum creatinine level of the patient is more than or equal to 5.7mg/dl; or (c) the patient’s kidney function has deteriorated to the extent that, the patient requires treatment for kidney failure [36].

### Other variables

Information on participants’ demographic characteristics, cigarette smoking and medical history was obtained by using a standardized questionnaire administered by trained personnel. Age was defined as the age at the time of clinic examination. Height was measured in centimeters using a wall-mounted measuring tape and weight was measured in kilograms using a digital scale. Body mass index (BMI) is calculated as ratio of body weight (measured in kilograms) divided by the square of the body height (measured in meters). Samples of 40 mL of non-fasting venous blood samples were collected to measure serum lipids and glycated hemoglobin (HbA1C). All serum biochemistry tests were carried out at the National University Hospital Reference Laboratory. Blood pressure (BP) was measured with a digital automatic BP monitor (Dinamap model Pro Series DP110X-RW, 100V2; GE Medical Systems Information Technologies, Inc., Wauwatosa, Wisconsin). A third measurement was made if the systolic BP differed by more than 10mmHg or the diastolic BP by more than 5 mmHg. The mean between the two closest readings were then taken as the blood pressure for that individual. Hypertension was defined as systolic BP of ≥ 140 mm Hg, diastolic BP of ≥ 90 mm Hg, or self-reported previously diagnosed hypertension. Diabetes mellitus was defined as a casual plasma glucose measurement of ≥200 mg/dL (11.1 mmol/L), self-reported physician-diagnosed diabetes, use of glucose-lowering medication, or glycosylated haemoglobin (HbA1C) of ≥6.5%. For our final analysis, only participants with complete data were included (Participants with incomplete data: 653 [10.14%]).

### Statistical Analysis

All statistical analyses were performed using STATA statistical software (Version 10, StataCorp, College Station, Texas). Quantitative retinal vascular measures were analysed as binary (retinal arteriolar, venular caliber and retinal vascular fractal dimension: quartile 1 vs. quartiles 2–4) and continuous variables (per each standard deviation increase/decrease). Retinopathy was analysed as binary variable (absence vs. presence).

We compared baseline characteristics between those who were included and excluded (including prevalent ESRD cases) in our analysis by employing the chi-squared test or by t-test as appropriate. We also compared baseline characteristics between those with retinopathy and those without by employing the chi-squared test or by t-test as appropriate.

Logistic regression analysis was performed to calculate the odds ratio (OR) for the cross-sectional association between retinal microvascular parameters (retinopathy signs, caliber and fractal dimension) and prevalent ESRD, adjusted for age, gender and race. Further adjustments for eGFR, diabetes and hypertension were not performed due to the small sample size.

For the longitudinal analyses, those with prevalent ESRD at baseline (n = 21) were excluded. Cox regression analysis was performed to calculate the hazard ratio (HR) for incident ESRD, initially adjusted for age, gender, race and additionally for hypertension, diabetes and eGFR. A p-value of <0.05 was considered to be statistically significant. Additionally, we performed a stratified analysis to determine whether the observed association between presence of retinopathy and incident ESRD is affected by diabetic status.

## Results

Table 1 shows the baseline characteristics of those who were included and excluded from the data analysis. Participants who were excluded from the data analysis were older, had higher levels of systolic and diastolic blood pressure, total cholesterol, HbA1c, but lower levels of eGFR. Participants who were excluded were also more likely to be hypertensive, diabetic but less likely to be smokers. A total of 5763 participants with gradable retinal photographs and complete data were included for the analysis. 21 (0.36%) prevalent ESRD cases were identified. During a median follow-up of 4.3 years (baseline examination [2004 to 2006] and 31 December 2011), 33 (0.57%) participants developed ESRD. Of these 33 participants who developed incident ESRD, 27 (87.8%) were diabetic. Table 2 shows the baseline characteristics of the study population between those with retinopathy and those without, among the participants free of prevalent ESRD. Participants who had retinopathy were older, had higher levels of BMI, systolic and diastolic BP, HbA1c, but lower levels of HDL cholesterol and eGFR. Participants with retinopathy were also more likely to be hypertensive and diabetic.

**Table 1:** Baseline characteristics comparing between those who were included to those who were excluded from the study.

	Included (N = 5763)	Excluded (N = 674)	p-value*
	Mean (SD) or n (%)	Mean (SD) or n (%)	
Age, years	55.12 (10.01)	64.41 (10.78)	**<0.001**
Gender, male	2811 (48.78)	319 (47.33)	0.477
Body mass index, kg/m^2^	25.40 (4.83)	25.13 (4.91)	0.168
Systolic blood pressure, mmHg	139.81 (22.47)	154.49 (25.73)	**<0.001**
Diastolic blood pressure, mmHg	79.35 (10.90)	80.31 (11.90)	**0.033**
Total cholesterol, mmol/L	5.45 (1.05)	5.64 (1.25)	**<0.001**
HDL cholesterol, mmol/L	1.37 (0.34)	1.38 (0.35)	0.293
HbA1c, %	6.29 (1.36)	6.49 (1.52)	**0.001**
Hypertension	3181 (55.21)	530 (78.64)	**<0.001**
Diabetes	1935 (33.58)	323 (47.92)	**<0.001**
Current smoking,	966 (16.78)	78 (11.66)	**0.001**
Estimated glomerular filtration rate, mL/min/1.73m^2^	77.30 (18.90)	67.32 (20.32)	**<0.001**
Retinal arteriolar caliber, μm	141.05 (15.12)	139.33 (17.95)	**0.047**
Retinal venular caliber, μm	219.27 (21.49)	218.43 (24.80)	0.493
Retinal vascular fractal dimension	1.45 (0.025)	1.41 (0.0675)	**<0.001**
Any retinopathy	625 (10.85)	71 (12.07)	0.363

* p-value for differences between those who were included to those who were excluded, by t-test or chi-square test as appropriate

**Table 2:** Baseline characteristics comparing between those with retinopathy and those without.

	With Retinopathy (N = 625)	Without Retinopathy (N = 5138)	p-value*
	Mean (SD) or n (%)	Mean (SD) or n (%)	
Age, years	57.75 (10.06)	54.80 (9.95)	**<0.001**
Gender, male	315 (50.40)	2496 (48.58)	0.390
Body mass index, kg/m^2^	26.14 (4.82)	25.31 (4.83)	**0.038**
Systolic blood pressure, mmHg	149.08 (25.18)	138.68 (21.85)	**<0.001**
Diastolic blood pressure, mmHg	80.46 (11.52)	79.21 (10.81)	**0.007**
Total cholesterol, mmol/L	5.38 (1.18)	5.46 (1.04)	0.103
HDL cholesterol, mmol/L	1.30 (0.31)	1.38 (0.34)	**<0.001**
HbA1c, %	7.43 (2.05)	6.15 (1.17)	**<0.001**
Hypertension	447 (71.52)	2734 (53.22)	**<0.001**
Diabetes	290 (62.40)	1545 (30.07)	**<0.001**
Current smoking	102 (16.35)	864 (16.83)	0.759
estimated Glomerular Filtration Rate, mL/min/1.73m^2^	75.93 (22.06)	81.84 (17.88)	**<0.001**
Retinal arteriolar caliber, μm	142.26 (15.45)	140.90 (15.08)	**0.033**
Retinal venular caliber, μm	224.73 (23.06)	218.85 (21.25)	**<0.001**
Retinal vascular fractal dimension	1.45 (0.026)	1.45 (0.025)	0.127

* p-value for differences between those with Retinopathy and those without, by t-test or chi-square test as appropriate

Table 3 shows the association between retinal vascular measures and prevalent ESRD. In multivariate analysis, after adjusting for age, gender and race, presence of retinopathy was found to be positively associated with prevalent ESRD (OR, 3.21, 95% CI, 1.28 to 8.05). Table 4 shows the association between retinal vascular measures and risk of incident ESRD. In the Cox proportional-hazards regression model, after adjusting for age, gender and race, persons with retinal arteriolar caliber narrowing (HR, 1.39, 95% CI, 1.02 to 1.91, per SD decrease) and presence of retinopathy (HR, 7.90, 95% 3.97 to 15.70) at baseline were more likely to develop ESRD. The association between presence of retinopathy (HR, 2.51, 95% 1.14 to 5.54) at baseline and risk of ESRD persisted after further adjusting for hypertension, diabetes and eGFR. However, the association between retinal arteriolar narrowing and risk of ESRD diminished after further adjusting for hypertension, diabetes and eGFR. Retinal venular vessel widening and fractal dimension at baseline were not significantly associated with incident ESRD.

**Table 3:** Relation of retinal vascular caliber, retinal vascular fractal dimension and retinopathy signs to prevalent end stage renal disease.

	Model 1			
	No at risk	Incident n (%)	OR (95% CI)	p-value
Retinal arteriolar caliber, μm				
Quartile 1 (82.89–131.63)	1446	5 (0.41)	0.97 (0.37, 2.54)	0.953
Quartiles 2–4 (131.64–206.31)	4338	16 (0.37)	**Ref**	
per SD decrease (15.13)	5784	21 (0.36)	1.10 (0.73, 1.67)	0.646
Retinal venular caliber, μm				
Quartile 1 (104.03–205.62)	1444	8 (0.55)	1.54 (0.63, 3.78)	0.347
Quartiles 2–4 (205.63–301)	4340	13 (0.30)	**Ref**	
per SD decrease (21.51)	5784	21 (0.36)	1.40 (0.93, 2.10)	0.102
Retinal vascular fractal dimension				
Quartiles 1 (1.17–1.43)	1446	10 (0.69)	1.62 (0.62, 4.25)	0.325
Quartile 2–4 (1.44–1.51)	4338	11 (0.25)	**Ref**	
per SD decrease (0.025)	5784	21 (0.36)	1.23 (0.84, 1.80)	0.292
Presence of retinopathy				
No	5152	14 (0.27)	**Ref**	
Yes	632	7 (1.11)	**3.21 (1.28, 8.05)**	**<0.001**

OR: Odds ratio; Model 1: adjusted for age, gender, race

**Table 4:** Relation of retinal vascular caliber, retinal vascular fractal dimension and retinopathy signs to risk of end stage renal disease.

	Model 1				Model 2			
	No at risk	Incident n (%)	HR (95% CI)	p-value	No at risk	Incident n (%)	HR(95% CI)	p-value
Retinal arteriolar caliber, μm								
Quartile 1 (82.89–131.63)	1440	16 (1.11)	1.98 (0.99, 3.97)	0.054	1440	16 (1.11)	1.39 (0.68, 2.81)	0.364
Quartiles 2–4 (131.64–206.31)	4323	17 (0.39)	**Ref**		4322	17 (0.39)	**Ref**	
per SD decrease (15.12)	5763	33 (0.57)	**1.39 (1.02, 1.91)**	**0.039**	5762	33 (0.57)	1.32 (0.92, 1.89)	0.134
Retinal venular caliber, μm								
Quartile 1 (104.03–205.64)	1440	15 (1.04)	1.79 (0.90, 3.60)	0.099	1440	15 (1.04)	1.31 (0.64, 2.69)	0.460
Quartiles 2–4 (205.63–301)	4323	18 (0.42)	**Ref**		4322	18 (0.42)	**Ref**	
per SD decrease (21.49)	5763	33 (0.57)	1.22 (0.93, 1.60)	0.258	5762	33 (0.57)	1.17 (0.83 to 1.64)	0.368
Retinal vascular fractal dimension								
Quartiles 1 (1.17–1.43)	1444	9 (0.63)	1.62 (0.76, 3.45)	0.214	1440	9 (0.63)	1.18 (0.55, 2.50)	0.673
Quartile 2–4 (1.44–1.51)	4319	24 (0.56)	**Ref**		4322	24 (0.56)	**Ref**	
per SD decrease (0.025)	5763	33 (0.57)	1.17 (0.72 to 1.92)	0.154	5762	33 (0.57)	0.99 (0.74 to 1.34)	0.971
Presence of retinopathy								
No	5138	15 (0.29)	**Ref**		5137	15 (0.29)	**Ref**	
Yes	625	18 (2.88)	**7.90 (3.97, 15.70)**	**<0.001**	625	18 (2.88)	**2.51 (1.14, 5.54)**	**0.022**

HR = hazard ratio; SD = standard deviation

Model 1: adjusted for age, gender, race; Model 2: additionally adjusted for hypertension, diabetes and eGFR

Table 5 shows the association between retinal vascular measures and incident ESRD stratified by diabetic status. After adjusting for age, gender, race and hypertensive status, eGFR and HbA1C, presence of retinopathy was associated with increased risk of ESRD development in participants with diabetes (HR, 2.60, 95% CI, 1.01, 6.66). This association was not present in participants without diabetes (HR, 1.65, 95% CI, 0.14, 18.98).

**Table 5:** Relation of retinal vascular caliber, retinal vascular fractal dimension and retinopathy signs to risk of end stage renal disease.

	Model 1				Model 2			
	No at risk	Incident n (%)	HR (95% CI)	p-value	No at risk	Incident n (%)	HR(95% CI)	p-value
Subject without diabetes mellitus								
Retinal arteriolar caliber, μm								
Quartile 1 (82.89–131.63)	964	4 (0.41)	3.33 (0.59, 18.78)	0.173	964	4 (0.41)	2.63 (0.35, 19.87)	0.350
Quartiles 2–4 (131.64–206.31)	2864	2 (0.07)	**Ref**		2864	2 (0.07)	**Ref**	
per SD decrease (15.03)	3828	6 (0.16)	1.41 (0.68, 2.96)	0.357	3828	6 (0.16)	1.68 (0.61, 4.65)	0.318
Retinal venular caliber, μm								
Quartile 1 (104.03–205.64)	953	2 (0.21)	0.89 (0.16, 5.05)	0.897	953	2 (0.21)	0.68 (0.10, 4.39)	0.684
Quartiles 2–4 (205.65–301)	2875	4 (0.14)	**Ref**		2875	4 (0.14)	**Ref**	
per SD decrease (21.06)	3828	6 (0.16)	0.73 (0.37, 1.45)	0.367	3828	6 (0.16)	0.57 (0.25, 1.32)	0.190
Retinal vascular fractal dimension								
Quartiles 1 (1.17–1.43)	864	4 (0.46)	2.29 (0.36, 14.78)	0.383	864	4 (0.46)	2.77 (0.38, 20.07)	0.313
Quartile 2–4 (1.44–1.51)	2964	2 (0.07)	**Ref**		2964	2 (0.07)	**Ref**	
per SD decrease (0.025)	3828	6 (0.16)	1.19 (0.63, 2.23)	0.592	3828	6 (0.16)	1.33 (0.69, 2.58)	0.386
Presence of retinopathy								
No	3593	5 (0.14)	**Ref**		3593	5 (0.14)	**Ref**	
Yes	234	1 (0.43)	2.57 (0.30, 22.11)	0.389	234	1 (0.43)	1.65 (0.14, 18.98)	0.689
Subject with diabetes mellitus								
Retinal arteriolar caliber, μm								
Quartile 1 (88.89–131.63)	476	12 (2.52)	2.00 (0.92, 4.33)	0.078	476	12 (2.52)	1.24 (0.55, 2.81)	0.608
Quartiles 2–4 (131.68–204.32)	1459	15 (1.03)	**Ref**		1459	15 (1.03)	**Ref**	
per SD decrease (15.31)	1935	27 (1.40)	1.46 (1.03, 2.07)	0.036	1935	27 (1.40)	1.23 (0.80, 1.89)	0.343
Retinal venular caliber, μm								
Quartile 1 (128.44–205.6)	487	13 (2.67)	2.25 (1.05 4.83)	0.038	487	13 (2.67)	1.35 (0.58, 3.17)	0.485
Quartiles 2–4 (205.75–289.61)	1448	14 (0.97)	**Ref**		1448	14 (0.97)	**Ref**	
per SD decrease (22.29)	1935	27 (1.40)	1.42 (1.01, 2.01)	0.046	1935	27 (1.40)	1.31 (0.88, 1.94)	0.177
Retinal vascular fractal dimension								
Quartile 1 (82.89–131.63)	573	14 (2.44)	1.53 (0.67, 3.49)	0.317	573	14 (2.44)	1.01 (0.44, 2.37)	0.973
Quartiles 2–4 (82.89–131.63)	1362	13 (0.95)	**Ref**		1362	13 (0.95)	**Ref**	
per SD decrease (0.025)	1935	27 (1.40)	1.24 (0.91, 1.69)	0.175	1935	27 (1.40)	0.95 (0.68, 1.34)	0.785
Presence of retinopathy								
No	1545	10 (0.65)	**Ref**		1545	10 (0.65)	**Ref**	
Yes	390	**17 (4.36)**	**5.72 (2.61, 12.56)**	**<0.001**	390	17 (4.36)	**2.60 (1.01, 6.66)**	**0.047**

HR = hazard ratio; SD = standard deviation

Model 1: adjusted for age, gender, race; Model 2: additionally adjusted for hypertension, eGFR and HbA1C

## DISCUSSION

In this study, we demonstrated that the presence of retinopathy was related to both prevalent and incident ESRD in a multi-ethnic population.

With advancements in retinal photography, retinal vascular imaging has been proposed to be a non-invasive tool to objectively and reliably assess microangiopathic changes. Retinopathy reflects advanced stages of structural microvascular damage (e.g. breakdown of the blood—retina barrier) from age, inflammation, diabetes and hypertension; [39–41] which are also risk factors for the development of ESRD. [42–44] Inferring from our general analysis, it is plausible that microvascular changes (as indicated by presence of retinopathy) are indicative of pathophysiological changes that are associated with the development of ESRD. However, in a recent study done by Grunwald et al, [17] the authors reported that association between retinopathy and incident ESRD did not exist after further adjustments of 24-hour urine protein and eGFR. A possible explanation is that retinal microvasculature reflects the cumulative microvascular damage that contributes to the progression of ESRD but does not provide additional prognostic information beyond that provided by 24-hour urine protein and eGFR. [17]

In our stratified analysis for diabetics and non-diabetics, the presence of retinopathy was predictive of incident ESRD in diabetics but not in non-diabetics. Multiple studies have also reported a close link between diabetic retinopathy and renal impairment.[15,45–48] Specifically, a recent meta-analysis comprising of 2012 participants reported that diabetic retinopathy was predictive of diabetic nephropathy.[49] This may be partly explained by the similar pathogenesis of microvascular dysfunction in diabetic retinopathy and diabetic nephropathy, where both involves thickening of basement membrane of micro vessels and increased vessel leakage. [50] Clinically, this translates to presence of albuminuria in nephropathy [51] and presence of retinal exudates in diabetic retinopathy. [52] On the other hand, the non-significant association between retinopathy and incident ESRD in non-diabetics could be due to the small number of incident ESRD in this subgroup. Hence, further studies are warranted to confirm this finding.

Retinal arteriolar narrowing has been hypothesized to represent a dysregulation of the renin-angiotensin and endothelial systems. [53,54] In addition, endothelin, a peptide secreted by vascular endothelial cells and a potent vasoconstrictor, has been hypothesized to be associated with sclerotic renal changes and progression of ESRD. [55] Cross-sectional studies have consistently demonstrated the association between retinal arteriolar narrowing and microalbuminuria and renal impairment. [6,12] Similarly, in the MESA study, Yau et al concluded that retinal arteriolar narrowing predicts risk of stage 3 CKD in whites. However, the association between retinal arteriolar narrowing and incident renal impairment was not replicated in other studies. [14] [16] [17] Despite shorter follow-up period in our study (4.3 years) compared to Beaver dam CKD study (15 years), [16] we similarly did not observe any significant associations between retinal arteriolar narrowing and incident ESRD. It is possible that although retinal arteriolar narrowing and chronic kidney disease share similar pathophysiological mechanism, they may not be causally related, thus explaining the lack of prospective association. [16]

We further explored the relationship of retinal venular caliber and retinal vascular fractal dimension with incident ESRD. Sng et al have previously reported that suboptimal retinal vascular fractal dimension (the lowest and highest quintiles) was associated with an increased prevalence of CKD [defined as eGFR < 60 mL/min/1.73 m^2^], after adjusting for age, systolic blood pressure, diabetes and other risk factors in a cross sectional study (the Singapore Prospective Study Program, SP2). [10] While it has been shown by Sng et al [10] that retinal fractal dimension was cross-sectionally associated with CKD, we did not observe a significant prospective association between retinal fractal dimension and ESRD. A possible explanation could be that changes in retinal fractal dimension may not be an early indicator of deviation from normal microvasculature associated with ESRD development. We also did not observe any significant association between retinal venular caliber and incident ESRD.

The strengths of our study include its population-based sample, quantitative and masked evaluation of retinal vessel diameters, standardized measurement of renal function, and the availability of information on potential confounding factors. Several limitations of this study should be addressed. First, because of our small sample size of incident ESRD cases, we were unable to draw meaningful conclusion from our stratified analysis. As such, we are unable to confidently conclude that presence of retinopathy was not associated with increased risk of ESRD development in participants without diabetes in the current study. Further studies will be needed to confirm this exploratory finding. Second, as albuminuria data was not available, we could not include it into our multivariable model. Third, insufficient statistical power due to a small sample size of incident ESRD cases may also explain the lack of association between other retinal vascular parameters and incident ESRD.

In summary, we demonstrated that the presence of retinopathy was related to both prevalent and incident ESRD in Asians. Specifically, diabetic retinopathy signs are related to an increased risk of ESRD. Our findings may provide evidence that presence of retinopathy reflect early subclinical damage in the renal microvasculature that is subsequently associated with development of renal disease.

## Supporting Information

S1 Table Longitudinal population-based studies examining the associations between retinal vascular changes with incident renal impairment outcome. (DOCX)
