# Supplementary material for: Retinal Microvascular Abnormalities and Risk of Renal Failure in Asian Populations
Source: PLoS One. 2015 Feb 6;10(2):e0118076. doi: 10.1371/journal.pone.0118076 (PMC4320082; doi:10.1371/journal.pone.0118076)
Supplement: S1 Table — (DOCX) [file pone.0118076.s001.docx]

| **S1:** Longitudinal population-based studies examining the associations between retinal vascular changes with incident renal impairment outcome. | | | | |
| --- | --- | --- | --- | --- |
| **Author, References and Study Population** | **Sample size (follow up period)** | **Age/Race** | **Incident renal impairment outcome** | **Results** |
| Grunwald JE et al [[17](#_ENREF_17)], The CRIC study | n = 1852  (2.3 years) | 22-72 years/ Whites & Blacks | Incident ESRD: initiation of chronic dialysis therapy and kidney transplantation | Presence of retinopathy, wider arterioles and venules are not associated with incident ESRD. Larger A/V ratio is found to be associated with incident ESRD. |
| Yau JW et al [13], Multi-Ethnic Study of Atherosclerosis | n = 6,814  (4.8 years) | 45-84 yrs/ African Americans, Chinese, Hispanics, Whites | CKD stage 3 : development of eGFR <60 mL/min/1.73 m^2^ at visit 3 and 4 plus an annual decrease in eGFR >1 mL/min/1.73 m^2^ | Retinal arteriolar narrowing was associated with incident CKD stage 3 in whites only. |
| Edwards MS et al [14], Cardiovascular Health Study | n = 1,394  (4 years) | > 65 yrs/ African, Americans, and Whites | 0.3 mg/dL increase serum creatinine level or 20% or greater decline in eGFR | Presence of retinopathy was associated with developing renal impairment. |
| Wong TY et al [15], Atherosclerosis Risk in Communities Study | n = 10,056  (6 years) | 45-64 yrs/ Black, Whites | Increased in serum creatinine of at least 0.4 mg/dl or a death or hospitalization as a result of chronic kidney disease | Presence of retinopathy, arterio-venous ratio and arteriovenous nicking were associated with developing renal impairment. |
| Sabanayagam C et al [16], Beaver Dam CKD study | n = 3,199 (15 years) | 43–84 yrs/ Caucasians | eGFR <60 mL/min/1.73 m^2^ accompanied by a 25% decrease in eGFR | Retinal arteriolar and venular calibres were not associated with incident CKD**.** |
| CKD-EPI: CKD Epidemiology Collaboration equation; eGFR: estimated glomerular filtration rates; ACE: angiotensin-converting enzyme, BMI: body mass index; ACR: albumin-creatinine ratio; HR: Hazards Ratio | | | | |
